# Identification of a *LMNA* (c.646C>T) variant by whole-exome sequencing in combination with a dilated cardiomyopathy (DCM) related gene filter in a family with familiar DCM

**DOI:** 10.7555/JBR.32.20180003

**Published:** 2018-05-31

**Authors:** Liang Chen, Zhongyin Zhou, Huihe Lu, Ye Xie, Gang Li, Jianfei Huang, Dongsheng Zhao

**Affiliations:** 1. Department of Cardiology, The Second Affiliated Hospital of Nantong University, Nantong, Jiangsu 226001, China; 2. Department of Cardiology, The First Affiliated hospital of Hubei University of Technology, Xianning, Hubei 437600, China; 3. Department of Cardiology, The Third Affiliated Hospital of Nantong University, Nantong, Jiangsu 226001, China.

Dilated cardiomyopathy (DCM) is characterized by the dilated heart chambers and reduced systolic function in the absence of specific aetiology^[1]^. Approximately one third of DCM cases are hereditary. In recent years, DCM concomitant with arrhythmias and sudden death resulting from gene mutation has been widely reported^[2]^. In the current study, we report the identification of a mutation within lamin A (LMNA) (p.R216C) in a Han Chinese family with similar cardiac manifestations by using whole-exome sequencing in combination with a related gene filter and co-segregation analysis.

**Fig.1:**
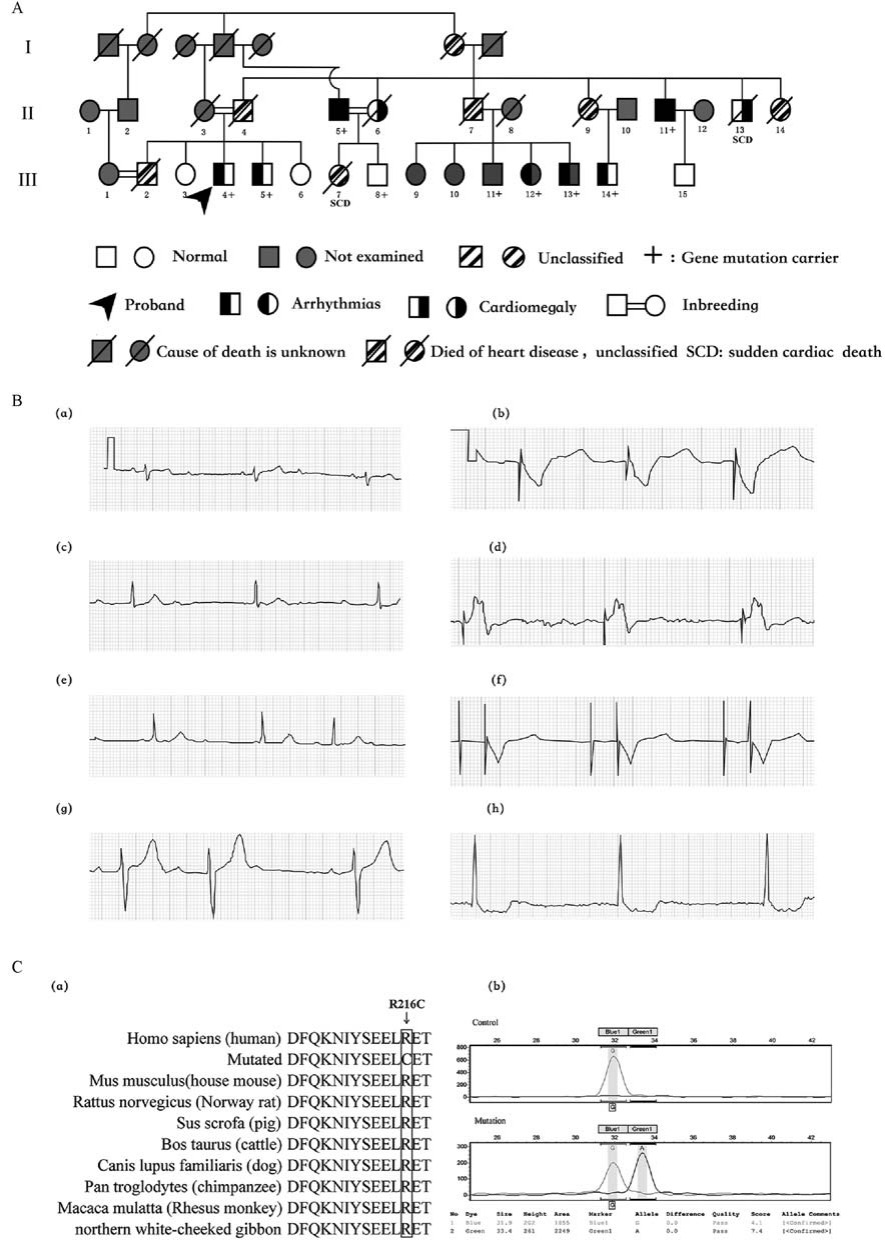
A LMNA variant in associatiation with familial dilated cardiomyopathy. A: The pedigree of the family of the proband. The proband (Ⅲ4), male and 53 years of age, complained of recurrent dizziness and was diagnosed with grade 3 atrioventricular block and had a pacemaker implantation. The left side of the blackening in the symbol denotes arrhythmia; the right denotes cardiomegaly and heart failure; blackening within both denotes both cardiomegaly/heart failure and arrhythmia. There are two members with sudden cardiac death ( 13\Ⅲ7) and three couples of consanguineous mating (Ⅱ3/4, Ⅱ5/6 and Ⅲ1/2). “+” denotes gene mutation carrier. B: The ECGs of the family members. The ECGs on the first visit Ⅱ5(a), Ⅱ11(c), Ⅲ4(e), Ⅲ13(g), Ⅲ14(h); and the follow-up visit Ⅱ5(b), Ⅱ11(d), Ⅲ4(f). C: Sequencing analysis of LMNA mutation (p. R216C). a: Alignment of multiple LMNA protein sequences across species. R216C affects amino acids that are located in the highly conserved amino acid region in different mammals. b: Sequencing results of the *LMNA* mutation. Sequence chromatogram indicates a C to T transition of nucleotide 646.

The study was approved by the local Ethics Committee of the First People^’^s Hospital of Nantong Jiangsu. A family from Jiangsu Province in eastern China was investigated with 24 hours-Holter monitoring and transthoracic echocardiography and the pedigree was built using Microsoft Office^[3]^. The information of the family members is shown in ***Fig. 1*** and ***Table 1***. Ⅲ4 (the proband, male, 53 years old) was hospitalized due to recurrent dizziness and amaurosis in 2012. The electrocardiogram showed grade 3 atrioventricular block (AVB), and a pacemaker was implanted. Both Ⅱ5 (male, 71 years old) and Ⅱ11 (male, 63 years old) had heart enlargement and pacemaker implantation for grade 3 AVB, and suffered from atrial fibrillation (2 and 5 years after pacemaker implantation). Ⅱ13 (male, 67 years old) has been suffering from refractory heart failure since 2003. In 2011, his condition deteriorated and he died suddenly. Ⅲ7, female, died suddenly at the age of 39 years after being awakened by telephone ringing. Ⅲ13 (male, 65 years old) and Ⅲ14 (male, 61 years old) both complained of fatigue and dizziness with their electrocardiogram showing grade 2 AVB (3: 2) and sinus stagnation respectively.

**Tab.1:** Summary of the main members of the family

Generation	Subject ID	Year of birth	Gender	ECG	Echocardiography			Medical history	Gene mutation
					LA (mm)	LV (mm)	LVEF (%)		
Ⅱ	5	1945	male	Ⅲ° AVB, AF	43	53	62	pacemaker implantation	+
Ⅱ	11	1953	male	Ⅲ° AVB, AF	39	56	54	pacemaker implantation	+
Ⅱ	13	1949	male	AF	56	69	28	DCM, sudden cardiac death	−
Ⅲ	4	1963	male	Ⅲ° AVB,	33	47	61	pacemaker implantation	+
Ⅲ	5	1967	male	Ⅰ° AVB	38	50	70	-	+
Ⅲ	7	-	female	-	-	-	-	sudden cardiac death	−
Ⅲ	12	1967	female	Ⅰ° AVB	35	49	63	-	+
Ⅲ	13	1953	male	Ⅱ° AVB	36	48	70	-	+
Ⅲ	14	1955	male	Junctional escape rhythm	44	51	60	-	+

LA: left atrium; LV: left ventricle; LVEF: left ventricular ejection fraction; AVB: auriculo-ventricular block; AF: atrial fibrillation; DCM: dilated cardiomyopathy.

Whole-exome sequencing in combination with a related gene filter and co-segregation analysis was used to detect the possible disease-causing mutation^[4]^. The variants within the whole-exome sequencing of the two affected members (Ⅱ:5 and Ⅱ:11) and the DCM-related variants^[2]^ were identified as suspected pathogenetic mutations. Then, with the measurement of SNaPshot^[5]^, segregation analysis was conducted both within and beyond the family. Two variants were found within the two samples and the DCM-related genes^[2]^. One is a synonymous mutation [SCN5A (E1061E)]. Co-segregation analysis of another [*LMNA* (c.646C>T p. R216C)] showed its presence in all affected family members and absence in the healthy ones except two (Ⅲ8, Ⅲ11). It was not found in either normal controls or sporadic cases outside the family. R216 is located in the highly conserved amino acid region in humans, pigs, cattle, dogs, and chimpanzees (***Fig. 1***). Three programs (Polyphen2, SIFT, and Mutation Taster) were used to analyze protein functions. The results showed that the p.R216C variants are probably damaging (0), damaging (0.004), and damaging (1), respectively.

*LMNA* mutations have been reported frequently as the causes of DCM concomitant with arrhythmias, often by autosomal dominant inheritance^[6^‒^8]^, which is consistent with our result. Ⅲ8 and Ⅲ11 are gene mutation carriers without cardiac abnormalities at present. In addition, the age dependence with the phenotype emergence has been reported in similar studies^[7^‒^9]^.

In summary, we report that the LMNA gene mutation (R216C) is related to a Han family’s structural and electrical cardiac disorders. Though this mutation was reported by van Riksingen* et al.* in 2013^[10]^, and in ClinVar (variation ID 200938), its pathogenicity remains controversial. So far as we know, there has not been a study of this mutation in China, and there is no related clinical or functional study.
